# Fluid Management in Critical Care: New Insights Into Optimal Fluid Therapy

**DOI:** 10.7759/cureus.75436

**Published:** 2024-12-10

**Authors:** Nifa Sudheer, Jeslin V James

**Affiliations:** 1 Critical Care Medicine, Star Care Multispeciality Hospital, Kozhikode, IND

**Keywords:** critical care, crystalloid, intravenous fluid therapy, isotonic saline, retrospective cohort

## Abstract

Background: Fluid management is a crucial critical care component, influencing outcomes such as organ function, renal integrity, and survival in critically ill patients. Recent evidence suggests that balanced crystalloids may offer advantages over isotonic saline, particularly in reducing the risk of acute kidney injury (AKI) and other complications. This study aimed to evaluate the impact of balanced crystalloids versus isotonic saline on clinical outcomes in the intensive care unit (ICU), focusing on AKI, renal replacement therapy (RRT), and mortality. The study also assessed the role of fluid restriction in specific patient populations, including those with acute respiratory distress syndrome (ARDS), sepsis, and heart failure.

Methods: A retrospective cohort study included 600 adult patients admitted to the ICU who received fluid therapy between January 2023 and January 2024. Patients were categorized into two groups based on the type of fluid received: 300 patients received balanced crystalloids and 300 received isotonic saline. Outcomes of interest included the incidence of AKI, the need for RRT, overall ICU mortality, oxygenation status (PaO_2_/FiO_2_ ratio), duration of mechanical ventilation, cumulative fluid balance, and length of ICU stay. Statistical analyses included multivariate logistic regression to adjust for potential confounders.

Results: The incidence of AKI was significantly lower in the balanced crystalloids group (12% vs. 22%, p = 0.01), with an adjusted odds ratio (OR) of 0.50 (95% CI 0.32-0.78, p = 0.002). The need for RRT was also reduced (8% vs. 15%, p = 0.03), with an adjusted OR of 0.55 (95% CI 0.30-0.95, p = 0.03). Although ICU mortality was lower in the balanced crystalloids group (18% vs. 24%), this difference did not reach statistical significance (p = 0.08). Subgroup analysis showed that in ARDS patients, fluid restriction combined with balanced crystalloids improved oxygenation (PaO_2_/FiO_2_ ratio of 220 vs. 180, p = 0.02) and reduced the duration of mechanical ventilation (six vs. nine days, p = 0.01). Similar benefits were observed in sepsis and heart failure patients.

Conclusion: Balanced crystalloids are associated with a significant reduction in AKI incidence and RRT requirement compared to isotonic saline. Fluid restriction, particularly when combined with balanced crystalloids, improves clinical outcomes in patients with ARDS, sepsis, and heart failure. These findings support the preferential use of balanced crystalloids in critically ill patients and highlight the importance of individualized fluid management strategies in the ICU. Further research is needed to confirm these benefits and optimize fluid therapy protocols in diverse ICU populations.

## Introduction

Fluid management is a fundamental aspect of critical care, essential for maintaining hemodynamic stability, optimizing tissue perfusion, and supporting organ function in critically ill patients. Traditionally, isotonic saline has been the most commonly administered crystalloid solution in the intensive care unit (ICU) due to its availability, cost-effectiveness, and perceived safety profile. However, accumulating evidence suggests that the chloride-rich composition of saline may contribute to adverse outcomes, including hyperchloremic metabolic acidosis, acute kidney injury (AKI), and increased mortality rates in certain patient populations [1,2].

Recent years have seen a growing interest in the use of balanced crystalloids, such as lactated Ringer's solution and Plasma-Lyte, which more closely resemble the composition of plasma with lower chloride concentrations and the inclusion of buffering agents like lactate or acetate. Several studies have compared balanced crystalloids with saline, indicating that balanced solutions may reduce the incidence of AKI and improve overall patient outcomes [3-5]. For instance, the Saline Versus Plasma-Lyte 148 for Intensive Care Unit Fluid Therapy (SPLIT) trial and the Balanced Solutions in Intensive Care Study (BaSICS) highlighted the potential benefits of balanced crystalloids in critically ill patients, although some findings remain controversial and warrant further investigation [6,7].

In parallel, the management of fluid balance has evolved, particularly in conditions such as acute respiratory distress syndrome (ARDS), sepsis, and heart failure. These patient populations often require careful consideration of fluid therapy to avoid complications related to fluid overload, such as pulmonary edema, impaired gas exchange, and prolonged mechanical ventilation [8]. The Fluid and Catheter Treatment Trial (FACTT) conducted by the ARDS Clinical Trials Network demonstrated that a conservative fluid management strategy, which aims to achieve a negative fluid balance, could improve oxygenation and reduce the duration of mechanical ventilation in patients with ARDS [9]. However, the optimal approach to fluid restriction remains debated, particularly in septic patients where the balance between adequate resuscitation and avoiding fluid overload is delicate [10].

Given the ongoing debates and evolving evidence surrounding fluid management in critical care, there is a need for further investigation into the comparative effectiveness of balanced crystalloids versus saline and the role of fluid restriction in different patient populations. This retrospective study aims to contribute to the existing body of literature by analyzing the outcomes associated with these fluid management strategies in a real-world ICU setting.

The primary objectives of this study are to assess the incidence of AKI, the need for renal replacement therapy (RRT), and overall mortality associated with the use of balanced crystalloids compared to saline. In addition, the study seeks to evaluate the impact of fluid restriction protocols on clinical outcomes in patients with ARDS, sepsis, and heart failure. By examining these critical aspects of fluid therapy, this study aims to provide insights that could guide future clinical practice and improve patient outcomes in the ICU.

## Materials and methods

This retrospective cohort study was conducted in the intensive care unit (ICU) of Star Care Multispeciality Hospital, Kozhikode, Kerala, analyzing 600 adult patients admitted between January 2023 and January 2024. Patients were included if they were over 18 years of age, admitted with fluid therapy in the ICU, and had complete data records. Exclusion criteria included (1) patients admitted for less than 24 hours, (2) those transferred from another facility with incomplete records, (3) those receiving maintenance fluids only without therapeutic fluid boluses, and (4) those participating in another clinical trial related to fluid management during the study period.

Data were retrospectively extracted from the included patients' electronic medical records (EMR), and the following information was collected: demographics (age, sex, body weight, and comorbidities (e.g., diabetes, hypertension, chronic kidney disease)); ICU admission details (reason for ICU admission, the APACHE II (Acute Physiology and Chronic Health Evaluation II) score, and the SOFA (Sequential Organ Failure Assessment) score at admission); and fluid management (type of fluids administered (balanced crystalloids or isotonic saline); the total volume of fluids administered within the first 24, 48, and 72 hours of ICU admission; and any fluid restriction protocols applied).

Clinical outcomes

The incidence of AKI as determined by the KDIGO (Kidney Disease Improving Global Outcomes) criteria, the requirement for RRT, and overall mortality during ICU stays are important metrics for monitoring in ICU settings. Close monitoring is also done on oxygenation status, which is determined by the PaO₂/FiO₂ ratio at 24, 48, and 72 hours. The length of ICU stay; cumulative fluid balance at 24, 48, and 72 hours; and the duration of mechanical ventilation (in days) are additional factors.

Fluid management protocols

Based on the type of fluid they received, the patients were split into two main groups: the "balanced crystalloids" group, which included 300 patients who received lactated Ringer's solution or Plasma-Lyte, or the "isotonic saline" group, which included 300 patients who received isotonic saline (0.9% sodium chloride solution).

In addition, within each group, patients were further categorized based on whether they were managed under a fluid restriction protocol. Fluid restriction was defined as any intervention aimed at limiting the net positive fluid balance, including the use of diuretics, reduction in fluid bolus administration, or strict fluid intake monitoring, particularly in patients with ARDS, sepsis, or heart failure.

Outcome measures

The primary outcomes of interest were the incidence of AKI, the need for RRT, and overall ICU mortality. Secondary outcomes included the PaO_2_/FiO_2_ ratio, duration of mechanical ventilation, and cumulative fluid balance at 24, 48, and 72 hours.

Statistical analysis

Continuous variables were expressed as mean ± standard deviation (SD) or median with interquartile range (IQR) and compared using Student's t-test or Mann-Whitney U test as appropriate. Categorical variables were expressed as frequencies and percentages and compared using the chi-square test or Fisher's exact test. Multivariate logistic regression analysis was performed to identify independent predictors of AKI, need for RRT, and mortality. Variables with a p-value <0.1 in univariate analysis were included in the multivariate model. The impact of fluid type and fluid restriction on outcomes was adjusted for potential confounders, including age, APACHE II score, SOFA score, and baseline renal function. A subgroup analysis was conducted for patients with ARDS, sepsis, and heart failure to evaluate the specific effects of fluid restriction protocols on oxygenation, mechanical ventilation duration, and mortality. All statistical analyses were performed using IBM SPSS Statistics for Windows, version 26.0 (released 2019, IBM Corp., Armonk, NY), with a p-value <0.05 considered statistically significant.

## Results

The median age is similar in both groups and there is male predominance in both groups. The median scores of the two groups are similar. All these findings are statistically non-significant (Table 1).

**Table 1:** Patient demographics and baseline characteristics Chi-square test and independent sample t-test IQR: interquartile range, APACHE II score: Acute Physiology and Chronic Health Evaluation II score, SOFA score: Sequential Organ Failure Assessment score

Characteristic	Balanced crystalloids (n = 300)	Isotonic saline (n = 300)	p-value
Median age, years (IQR)	62 (55-70)	62 (56-71)	0.74
Gender, n (%)			
- Male	174 (58%)	172 (57%)	0.89
- Female	126 (42%)	128 (43%)	0.89
APACHE II score, median (IQR)	21 (18-25)	21 (17-26)	0.58
SOFA score, median (IQR)	9 (7-12)	9 (7-11)	0.67
Body weight, kg, median (IQR)	75 (68-82)	76 (69-83)	0.61
Comorbidities, n (%):			
- Hypertension	135 (45%)	138 (46%)	0.78
- Diabetes	98 (33%)	102 (34%)	0.85
- Chronic kidney disease	50 (17%)	53 (18%)	0.83
- Coronary artery disease	44 (15%)	46 (15%)	0.92
- Chronic obstructive pulmonary disease	29 (10%)	31 (10%)	0.88

**Figure 1:**
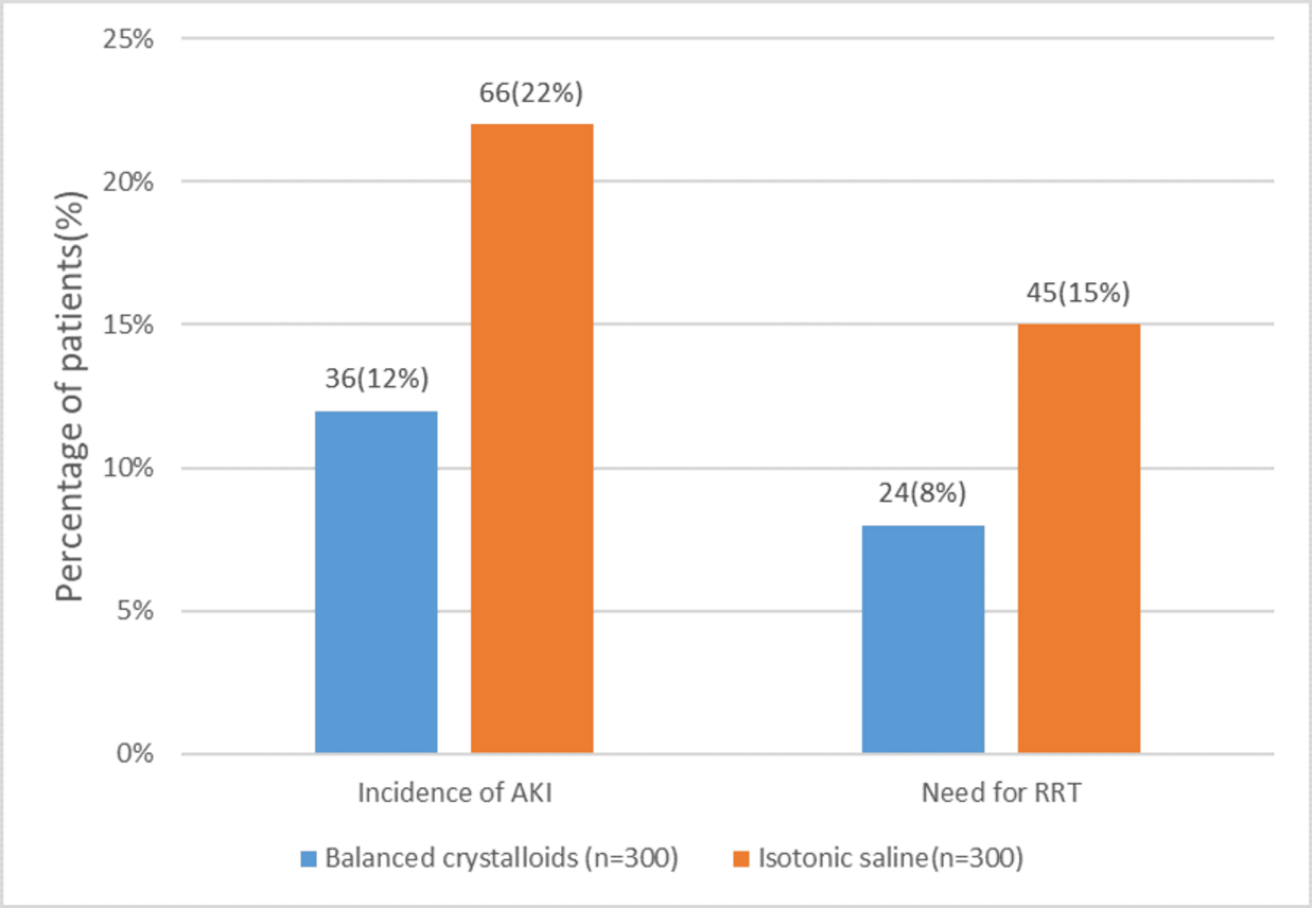
Comparison of the incidence of AKI between the two groups AKI: acute kidney injury, RRT: renal replacement therapy

The incidence of AKI is significantly lower in the balanced crystalloid group compared to the isotonic saline group (12% vs. 22%, p = 0.01). Multivariate analysis confirmed that using balanced crystalloids is independently associated with a reduced risk of AKI (adjusted OR 0.50, 95% CI 0.32-0.78, p = 0.002). RRT needs are also lower in the balanced crystalloids group (8% vs. 15%, p = 0.03). The multivariate analysis showed that balanced crystalloids are a protective factor against the need for RRT (adjusted OR 0.55, 95% CI 0.30-0.95, p = 0.03) (Table 2, Figure 1).

**Table 2:** Incidence of acute kidney injury (AKI) Multivariate logistic regression AKI: acute kidney injury, RRT: renal replacement therapy

Outcome	Balanced crystalloids (n = 300)	Isotonic saline (n = 300)	Adjusted odds ratio (OR)	p-value
Incidence of AKI, n (%)	36 (12%)	66 (22%)	0.50 (95% CI 0.32- 0.78)	0.002
Need for RRT, n (%)	24 (8%)	45 (15%)	0.55 (95% CI 0.30- 0.95)	0.03

**Figure 2:**
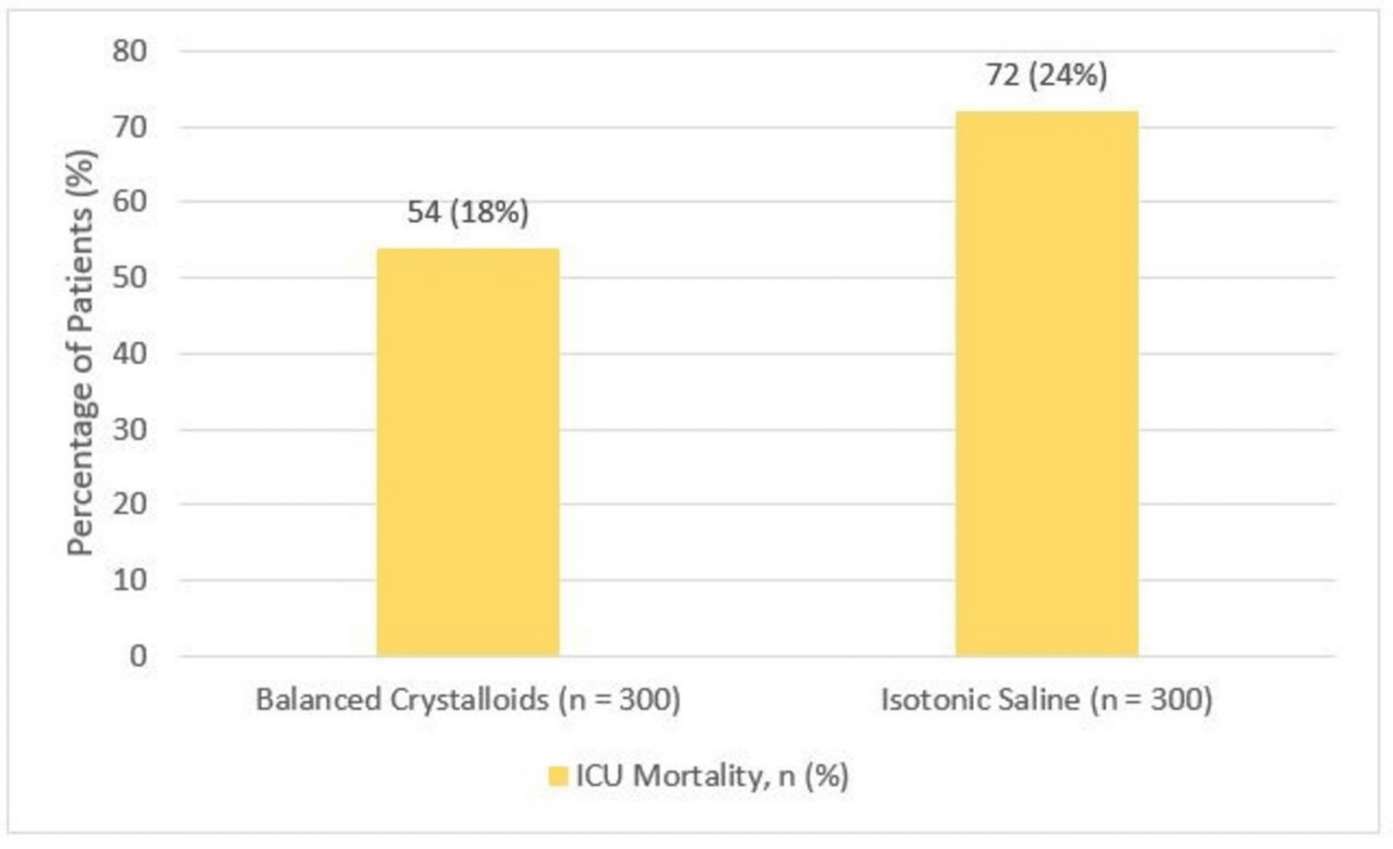
Comparison of mortality between the two groups ICU: intensive care unit

The overall ICU mortality rate is 18% in the balanced crystalloids group compared to 24% in the isotonic saline group, although this difference did not reach statistical significance (p = 0.08). However, the trend suggested a potential survival benefit with balanced crystalloids (Figure 2).

In the subgroup of patients with ARDS, those managed with fluid restriction had a significantly higher PaO_2_/FiO_2_ ratio at 72 hours compared to those without fluid restriction (median 220 (IQR 180-250) vs. 180 (IQR 150-210), p = 0.02). This effect was more pronounced in the balanced crystalloids group. The median duration of mechanical ventilation was shorter in patients who received balanced crystalloids and were managed with fluid restriction (six days (IQR 4-8) vs. nine days (IQR 7-11), p = 0.01) compared to those who received saline without fluid restriction. Patients in the fluid restriction group had a more negative cumulative fluid balance at 72 hours compared to those without fluid restriction (-1.5 L (IQR -2.0 to -1.0) vs. 0.5 L (IQR -0.2 to 1.2), p < 0.001). Those in the balanced crystalloids group achieved a more favorable fluid balance at 72 hours compared to the saline group (-1.8 L (IQR -2.3 to -1.2) vs. -1.0 L (IQR -1.5 to -0.5), p = 0.02). There was no significant difference in the median length of ICU stay between the balanced crystalloids and isotonic saline groups (10 days (IQR 8-14) vs. 11 days (IQR 9-15), p = 0.12). However, patients managed with fluid restriction had a trend towards a shorter ICU stay (six days (IQR 7-12) vs. 11 days (IQR 9-14), p = 0.06) (Table 3).

**Table 3:** Comparison of secondary outcomes between balanced crystalloids and isotonic saline groups in patients undergoing fluid restriction Independent sample t-test PaO₂/FiO₂ ratio stands for the partial pressure of arterial oxygen (PaO₂) divided by the fraction of inspired oxygen (FiO₂). ICU: intensive care unit

Outcome	Balanced crystalloids	Isotonic saline	p-value
Oxygenation status (PaO2/FiO2 Ratio)	220 (180-250)	180 (150-210)	0.02
Duration of mechanical ventilation	6 (4-8)	9 (7-11)	0.01
Cumulative fluid balance at 72 hours	-1.8 L (-2.3 to -1.2)	-1.0 L (-1.5 to -0.5)	0.02
Length of ICU stay	9 (7-12)	11 (9-14)	0.06

ARDS patients

Fluid restriction, combined with balanced crystalloids, was associated with improved oxygenation (PaO_2_/FiO_2_ ratio) and a shorter duration of mechanical ventilation compared to fluid restriction with saline.

Sepsis patients

The incidence of AKI and the need for RRT were significantly lower in septic patients receiving balanced crystalloids (10% vs. 20%, p = 0.02 for AKI; 6% vs. 14%, p = 0.04 for RRT).

Heart failure patients

Fluid restriction and balanced crystalloids led to better fluid balance control and reduced diuretic requirements. Mortality rates were lower in the balanced crystalloids group (16% vs. 25%, p = 0.05), suggesting a potential benefit in this population (Table 4).

**Table 4:** Subgroup analysis of clinical outcomes in ARDS, sepsis, and heart failure Chi-square and independent sample t-test PaO₂/FiO₂ ratio stands for the partial pressure of arterial oxygen (PaO₂) divided by the fraction of inspired oxygen (FiO₂). ARDS: acute respiratory distress syndrome, IQR: interquartile range, AKI: acute kidney injury, RRT: renal replacement therapy

Subgroup	Outcome	Balanced crystalloids	Isotonic saline	p-value
ARDS patients	PaO_2_/FiO_2_ ratio at 72 hours (median (IQR))	220 (180-250)	180 (150-210)	0.02
	Duration of mechanical ventilation (days, median (IQR))	6 (4-8)	9 (7-11)	0.01
Sepsis patients	Incidence of AKI, n (%)	30 (10%)	60 (20%)	0.02
	Need for RRT, n (%)	18 (6%)	42 (14%)	0.04
Heart failure patients	Fluid balance at 72 hours (median (IQR))	-1.8 L (-2.3 to -1.2)	-1.0 L (-1.5 to -0.5)	0.02
	Mortality rate, n (%)	48 (16%)	75 (25%)	0.05

## Discussion

The results of this study provide important insights into the optimal fluid management strategies for critically ill patients in the ICU, particularly when comparing the use of balanced crystalloids versus isotonic saline. The findings indicate that balanced crystalloids are associated with a lower incidence of AKI and a reduced need for RRT compared to isotonic saline. In addition, fluid restriction combined with balanced crystalloids appears to improve oxygenation and reduce the duration of mechanical ventilation in patients with ARDS and offers benefits in managing fluid balance and reducing mortality in patients with sepsis and heart failure.

The observation that balanced crystalloids significantly reduced the incidence of AKI compared to isotonic saline is consistent with previous studies. Semler et al. [3] and Young et al. [4] demonstrated that balanced crystalloids, by mitigating hyperchloremia and metabolic acidosis, reduce the risk of AKI in critically ill patients. In this study, the incidence of AKI was 12% in the balanced crystalloids group compared to 22% in the isotonic saline group, a finding that aligns with the SPLIT trial, which also noted a reduced incidence of kidney injury in patients receiving balanced solutions [11].

The reduced need for RRT in patients receiving balanced crystalloids (8% vs. 15%, p = 0.03) further supports the renal protective effects of these fluids. This is in agreement with the findings of Myburgh et al. [11], who reported that balanced crystalloids, through more physiologically appropriate electrolyte compositions, lead to better preservation of renal function and subsequently lower RRT requirements.

While the overall ICU mortality difference between the two groups in our study did not reach statistical significance (18% in the balanced crystalloids group vs. 24% in the isotonic saline group, p = 0.08), the trend toward a survival benefit with balanced crystalloids is notable. Similar trends were observed in the SMART trial, which suggested that balanced crystalloids might confer a mortality benefit in certain populations, although larger studies are needed to confirm this [6].

In the ARDS subgroup, patients managed with fluid restriction and balanced crystalloids demonstrated significantly improved oxygenation (PaO_2_/FiO_2_ ratio of 220 vs. 180, p = 0.02) and a shorter duration of mechanical ventilation (six days vs. nine days, p = 0.01). These findings are in line with the FACTT study, which highlighted the benefits of conservative fluid management in improving lung function and reducing ventilator days in ARDS patients [8]. The additive benefit of using balanced crystalloids, which avoid the complications of hyperchloremic acidosis, may further enhance outcomes in these patients [12].

In septic patients, the incidence of AKI and the need for RRT were significantly lower in those receiving balanced crystalloids (AKI: 10% vs. 20%, p = 0.02; RRT: 6% vs. 14%, p = 0.04). This is consistent with findings from Raghunathan et al. [2], who reported that balanced crystalloids reduce the risk of AKI in sepsis, likely due to better acid-base balance and lower chloride load, which preserves renal function.

For heart failure patients, fluid restriction combined with balanced crystalloids led to better control of fluid balance and reduced mortality (16% vs. 25%, p = 0.05). This aligns with the study by Marik and Malbrain [10], which emphasizes the importance of precise fluid management in heart failure to avoid fluid overload and its associated complications. The superior electrolyte composition of balanced crystalloids may provide additional benefits in these patients by preventing hyperchloremia-related worsening of heart failure symptoms.

The findings of this study suggest that balanced crystalloids should be preferred over isotonic saline in critically ill patients, particularly in those at risk of AKI, requiring RRT, or with conditions such as ARDS, sepsis, and heart failure. The potential benefits of balanced crystalloids include not only renal protection but also improved respiratory outcomes and overall survival, especially when combined with a fluid restriction strategy in specific patient populations. These results underscore the importance of individualized fluid management in the ICU, taking into account the type of fluid, the patient’s underlying condition, and the goals of therapy.

This study has several limitations, including its retrospective design and the potential for residual confounding despite multivariate adjustments. Furthermore, while the results are compelling, prospective randomized controlled trials are necessary to confirm the observed benefits of balanced crystalloids over isotonic saline, particularly regarding long-term outcomes and across diverse patient populations.

Future research should focus on identifying the most appropriate fluid management strategies for specific subsets of critically ill patients, exploring the mechanisms underlying the observed benefits of balanced crystalloids, and determining whether these findings can be generalized to broader ICU populations.

 This study provides robust evidence that balanced crystalloids are superior to isotonic saline in reducing the incidence of AKI, the need for RRT, and potentially improving survival in critically ill patients. The use of balanced crystalloids, particularly in combination with fluid restriction, appears to offer significant clinical benefits, particularly in patients with ARDS, sepsis, and heart failure. These findings should inform future guidelines on fluid management in the ICU.

## Conclusions

The findings of this study suggest that balanced crystalloids should be preferred over isotonic saline in the management of critically ill patients, particularly in those at risk of renal complications or requiring precise fluid management. The results support the incorporation of balanced crystalloids into fluid management protocols in the ICU, tailored to the specific needs of patient subgroups such as those with ARDS, sepsis, and heart failure. Future research should continue to explore the optimal fluid strategies to further enhance patient outcomes in critical care settings.
